# The comparison of serum TGF-beta levels and associated polymorphisms in patients with coronary artery ectasia and normal coronary artery

**DOI:** 10.1186/s43044-021-00153-w

**Published:** 2021-03-31

**Authors:** Özgür Selim Ser, Gökhan Çetinkal, Onur Kiliçarslan, Yalçın Dalgıç, Servet Batit, Kudret Keskin, Gulçin Özkara, Ezgi Irmak Aslan, Hülya Yilmaz Aydoğan, Ahmet Yıldız, Zerrin Yiğit

**Affiliations:** 1 Department of Cardiology, Institute of Cardiology, Istanbul University-Cerrahpasa, Haseki / Fatih, 34100 Istanbul, Turkey; 2 Department of Cardiology, Sisli Hamidiye Etfal Training and Research Hospital, Health Sciences University, Istanbul, Turkey; 3 Department of Molecular Medicine, Aziz Sancar Institute of Experimental Medicine, Istanbul University, Istanbul, Turkey

**Keywords:** TGF-β1, Polymorphism, Coronary artery ectasia

## Abstract

**Background:**

Coronary artery ectasia (CAE) is described as the enlargement of a coronary artery segment by 1.5 times or more, which is generally associated with the atherosclerotic process. Atherosclerotic changes lead to arterial remodeling result in CAE. In our study, we measured serum transforming growth factor (*TGF*)*-β1* levels, which have a protective role against atherosclerosis. Further, we aimed to assess the *TGF-β1* gene variants rs1800469 (–509C>T, c.−1347C>T) and rs1800470 (c.+29T>C, p.Pro10Leu, rs1982073), which might have an effect on TGF production. Overall, 2877 patients were screened including 56 patients with CAE and 44 patients with normal coronary arteries who were included in the study. Serum TGF-β1 levels were measured using ELISA and compared between two groups. Additionally, *TGF-β1* rs1800469 and rs1800470 gene variations were determined using TaqMan® SNP Genotyping Assays.

**Results:**

Serum TGF-β1 levels were significantly lower in patients with CAE than in controls (*p*=0.012). However, there was no difference in terms of the genotype and allele distributions of *TGF-β1* rs1800469 and rs1800470 polymorphisms. Serum TGF-β1 levels were higher in individuals carrying the *TGF-β1* rs1800470 G allele (GG+AG) than in individuals with normal homozygous AA genotype in the CAE group (*p*=0.012).

**Conclusion:**

Our findings suggest that lower serum TGF-β1 levels are associated with an increased risk for CAE development and that *TGF-β1* polymorphisms exert a protective effect. Furthermore, *TGF-*β1 rs1800470 G allele carriers were shown to have higher TGF-β1 levels in the CAE group. This suggests that having the G allele in the *TGF-β1* rs1800470 polymorphism could prevent CAE development.

## Background

Coronary artery ectasia (CAE) is described as the enlargement of an artery segment by 1.5 times or more compared with the adjacent normal segment. It is diagnosed angiographically, and it may be localized or diffused and observed in multiple branches. CEA, which is usually accompanied by coronary artery disease, can be congenital or acquired. Its frequency is reported to be around 1.4 to 5.3% based on studies that were conducted in Europe and the USA [1–4]. Both atherosclerosis and CAE share common risk factors such as male gender, hypertension, hyperlipidemia, smoking, and history of stroke [2, 5].

Pathophysiological findings change according to the underlying etiology in CAE patients. The etiology in half of the CAE patients is atherosclerosis. Atherosclerotic changes that occur in the tunica intima deteriorate the elastic composition of tunica media and lead to arterial remodeling, which results in CAE [4, 5].

The transforming growth factor (TGF)-β superfamily, which comprises TGF-β, activin, inhibin, growth and differentiation factors, and bone morphogenetic proteins, is a multifunction regulator that stimulates cell division, differentiation, organization, migration, and adhesion. TGF-β has the following three isotypes: TGF-β1, TGF-β2, and TGF-β3. TGF-β1 is the main effective isotype on the cardiovascular system [6, 7].

TGF-β1 plays a key role in the pathogenesis of many cardiac diseases such as hypertension, in-stent restenosis, atherosclerosis, left ventricular hypertrophy, and heart failure. TGF-β1 has a pleiotropic effect on cardiovascular cells [6, 8]. It has been confirmed to be a protective cytokine because it is involved in maintaining vessel wall integrity, reducing inflammation, and maintaining the extracellular matrix (ECM) content in atherosclerosis. If the protective effect of TGF-β1 is lost, the atherosclerosis process may accelerate. TGF-β also contributes to plaque stabilization by increasing collagen production [6, 8, 9].

Genetic polymorphism is the occurrence of two or more alleles of a gene in the region (locus) at a certain frequency in the same population. Polymorphisms are formed as a result of mutation. The most common type of genetic polymorphism among humans is single nucleotide polymorphisms, which are often called SNPs. Each SNP is an alteration of the DNA sequence of a nucleotide. Genetic polymorphism may also occur as a result of the addition or removal of DNA sequence of a nucleotide. Most polymorphisms are silent and do not alter gene function or expression, but a polymorphic variant of a gene may lead to abnormal expression or production of an abnormal form of the protein; this alteration can cause or be related to diseases.

Rs1800469 (T-509C) is a variation in the promoter region of the *TGF-β1* gene that affects gene transcriptional activity and serum TGF-β1 levels. Rs1800470 (Leu10Pro) is a missense polymorphism that is located in the coding sequence of the *TGF-β1* gene and leads to alterations in the amino acid sequence and TGF-β1 secretion from cells [10]. Both polymorphisms were found to be associated with coronary heart disease (CHD) complications in a large-scale meta-analysis study [11].

CAE is an inflammatory disease that develops in the background of atherosclerosis due to progressive destruction of collagen/elastin, and TGF-β1 plays a prominent role in inflammatory processes. While *TGF-β1* variations and plasma levels have been investigated alone or together in different diseases, to the best of our knowledge, there has been no investigation regarding the effect of *TGF-β1* variations on TGF-β1 levels in CAE disease. Moreover, in other populations, *TGF-β1* variations have not been examined enough except in thoracic and abdominal aortic aneurysm studies. Based on the hypothesis that TGF-β1 is a protective cytokine against atherosclerosis and that variations in the *TGF-β1* gene affect the pathogenesis of CAE, we determined the effects of *TGF-β1* rs1800469 and rs1800470 polymorphisms in the development of CAE.

## Methods

### Study population

In this prospective observational study, 2877 patients who underwent elective coronary angiography from January 2017 through December 2017 were evaluated, and among them, 56 patients with CAE were included in the study. The control group comprised 44 patients who were referred for CAG either due to a positive exercise test or typical ischemic symptoms and found to be free of coronary artery disease (normal coronary arteries). Exclusion criteria were serious previous psychiatric disorders, ≥50% occlusive coronary artery lesion with CAE, acute coronary syndromes, serious pulmonary hypertension, extremely high blood pressure (>180/110 mmHg) despite antihypertensive treatment, liver disease, history of stroke, myocardial infarction, previous cardiac surgery, collagenous disease, chronic renal disease, peripheral artery disease, or hyperthyroidism.

When we recruited the patients, in order to avoid the selection bias, we enrolled the first patient with normal coronary arteries, who was admitted after a patient with CAE, as the control.

Ethics board approval was obtained from the Clinical Research Ethics Committee and written, and informed consent was obtained from each participant. Those who were unable to cooperate or who did not comply with the study requirements were excluded from the study.

### Genotyping

Blood samples were collected into EDTA containing tubes and total DNA was isolated according to the protocol of a commercial kit (PureLink Genomic DNA Mini Kit, Cat. No: K182002, Thermo Fisher Scientific, USA). DNA concentration and purity were determined using a NanoDrop 2000 Spectrophotometer (Thermo Fisher Scientific). *TGF-β1* rs1800469 [−509 G>A (C>T)] (Assay ID: C_8708473_10) and rs1800470 [+869A>G (T>C)] (Assay ID: C_22272997_10) variations were detected by real-time PCR using ready-to-use TaqMan® SNP Genotyping Assays (40X) (Cat. No: 4351379, Thermo Fisher Scientific) in which one allele was labeled with FAM^TM^ and the other was labeled with VIC® fluorescent dyes. The PCR reaction mix was prepared according to TaqMan® Universal Master Mix II (UNG) (Cat. No: 4440038, Thermo Fisher Scientific) protocol and the Applied Biosystems 7500 Real-Time PCR Instrument (Thermo Fisher Scientific) was used for thermal cycling with the following conditions: 2 min at 50°C (pre-incubation), 2 min at 95°C (activation), 15 s at 95°C (denaturation), and 1 min at 60°C (binding/extension/reading) (40 cycles). Genotypes were automatically determined by the instrument in accordance with the FAM or VIC fluorescence signal produced during allele amplification.

### Detection of serum TGF-β1 levels

Blood samples were collected into plain tubes from participants who had fasted overnight and serum samples were obtained via centrifuged blood samples at room temperature for 10 min at 1500×*g.* Serum samples were immediately frozen at −80°C until the ELISA test was performed. Human TGF beta 1 Platinum ELISA (Cat. No: BMS249/4, Thermo Fisher Scientific), which is a commercial kit, was used to determine the *TGF-β1* levels in serum samples according to the test protocol. Absorbance (OD) measurements were performed using an ELISA plate reader (Multiskan™ GO Microplate Spectrophotometer) at a wavelength of 450 nm. After automatically calculating the standard concentrations (ng/mL for *TGF-β1*), serum concentrations were detected using the linear regression equation according to the optical density (OD) values.

### Statistical analysis

Statistical analyses were performed using SPSS 21 (SPSS Inc., Chicago, IL, USA). The data distribution was tested for normality using the Kolmogorov–Smirnov test. Allele frequencies were estimated by gene counting methods. Differences in the distribution of genotypes and alleles were assessed using the chi-square test. Mean values were compared between the patients and controls using an unpaired Student’s *t* test and an analysis of variance (ANOVA). The statistical significance limit was accepted as *p*<0.05. Diagnostic accuracy of TGF-β1 was evaluated using the area under receiver operating characteristic (ROC) curve (AUC) along with its 95% confidence interval (95% CI).

## Results

Demographic and clinical features of the study groups are shown in Table 1. Both groups had comparable mean ages. The ratio of male gender and the prevalence of hypertension and hyperlipidemia were higher in CAE patients than in controls. Biochemical and hematological parameters of the study groups are shown in Table 2. Hematocrit levels were higher and TGF-β1 levels were lower in CAE patients than in controls.

**Table 1:** Demographic and clinical features of study groups

	Study groups		***p*** value
	CAE patients (***n***=56)	Control patients (***n***=44)	
**Age (year)**	62.23±1.284	59.95± 1.79	0.292
**Gender (F/M)**	16/40	26/18	**0.002**
**EF (%)**	57.02±1.07	58.55±0.88	0.292
**HT (%)**	38 (67.9)	21 (47.7)	**0.042**
**DM (%)**	22 (39.3)	11 (25)	0.132
**Hyperlipidemia (%)**	33 (58.9)	13 (29.5)	**0.003**

Values were derived by using an independent-sample *t* test. The results were shown as mean ± SD and %. Values of *p* < 0.05 were considered statistically significant

*CAE* coronary artery ectasia, *DM* diabetes mellitus, *HT* hypertension, *EF* ejection fraction

**Table 2:** Biochemical and hematological parameters of study groups

	Study groups		***p*** value
	CAE patients (***n***=56)	Control patients (***n=***44)	
**TGF β1 (pg/ml)**	3250.40± 212.81	4851.21± 570.36	**0.012**
**Fasting glucose (mg/dl)**	119.23 ± 5.96	124.39± 10.25	0.649
**HbA1c (%)**	6.50± 0.18	6.16 ± 0.13	0.152
**ALT (SGPT) (U/L)**	27.43/3.49	22.52/2.29	0.271
**AST ( SGOT) (U/L)**	24.32/2.27	21.70/1.92	0.399
**BUN (mg/dl)**	15.82±0.52	16.82±1.01	0.355
**Creatine (mg/dl)**	0.92±0.03	0.86±0.03	0.290
**WBC (10**^**3**^**μl)**	8.44±0.35	7.932±0.37	0.322
**Hct (%)**	40.94±0.69	38.78±0.76	**0.040**
**Platelet (10**^**3**^**μl)**	239.27±9.15	249.95±10.49	0.444
**TSH (μu/ml)**	2.39±0.32	2.15±0.22	0.559

Values were derived by using an independent-sample *t* test. The results were shown as mean ± SD and %. Bold values of *p* < 0.05 were considered statistically significant

*CAE* coronary artery ectasia, *TGF β1* transforming growth factor beta 1, *HgA1c* hemoglobin A1c, *BUN* blood urea nitrogen, *Hgb* hemoglobin, *WBC* white blood cell, *Hct* hematocrit, *TSH* thyroid stimulating hormone

Genotype and allele distributions of *TGF-β1* rs1800469 and rs1800470 are shown in Table 3. There were no statistically significant differences between the study groups. In addition, genotype and allele distributions of *TGF-β1* rs1800469 and rs1800470 did not deviate from the Hardy–Weinberg equation (HWE) in CAE patients and healthy controls.

**Table 3:** *TGF β1* genotype and allele distributions of CAE and control patients

Genotype and allele distributions	CAE (***n***=56)	Control (***n***=44)
***TGF β1*rs1800469 genotypes (%)**		
**GG**	19 (33.9%)	16 (36.4%)
**AA**	12 (21.4%)	8 (18.2%)
**GA**	25 (44.6%)	20 (45.5%)
**rs1800469 Alleles (%)**		
**G**	63 (56.25%)	52 (59.09%)
**A**	49 (43.75%)	36 (40.91%)
**HWE**	*p=*0.486	*p=* 0.691
***TGF β1*rs1800470Genotypes (%)**		
**AA**	23 (41.1%)	13 (29.5%)
**GG**	10 (17.9%)	8 (18.2%)
**AG**	23 (41.1%)	23 (52.3%)
**rs1800470 Alleles (%)**		
**A**	69 (61.61%)	49 (55.68%)
**G**	43 (38.39%)	39 (44.32%)
**HWE**	*p*=0.324	*p*=0.694

The values in the table are given in *n* (%)

*CAE* coronary artery ectasia, *TGF β1* transforming growth factor beta1, *HWE* Hardy–Weinberg Equilibrium, *n* sample number

The association of *TGF-β1* rs1800469 (G>A) genotypes with clinical and biochemical features is shown in Table 4. We did not observe any statistically significant association when comparing the effects of *TGF-β1* rs1800469 genotypes on biochemical parameters in study groups. However, patients with the *TGF-β1* rs1800469 homozygous normal GG genotype were more likely to have decreased TGF-β1 level in the CAE group. This association showed the effect of *TGF-β1* rs1800469 on serum TGF-β1 levels as ascending levels in the following order: GG < GA < AA. However, this tendency was not observed in controls.

**Table 4:** Association between *TGF β1* rs1800469 genotype/alleles and clinical/hematological parameters in study groups

Parameters	Groups									
	CAE patients					Control patients				
	rs1800469Genotype			rs1800469Alleles		rs1800469Genotype			rs1800469Alleles	
	GG (***n***=19)	AA (***n***=12)	GA (***n***=25)	G Allele (***n***=44)	A Allele (***n***=37)	GG (***n***=16)	AA (***n***=8)	GA (***n***=20)	G Allele (***n***=36)	A Allele (***n***=28)
**Age (years)**	64.11±2.17	59.58±2.75	62.08±1.96	62.95±1.44	61.27±1.58	58.31±3.77	64.88±1.87	59.30±2.41	58.86±2.11	60.89±1.85
**TGF β1 (ng/ml)**	2841.76±361.86	3741.11±517.32	3360.43±302.65	3140.00±232.86	3467.50±259.02	4914.61±787.63	4580.00±757.07	4908.57±1121.77	4911.48±681.25	4810.00±805.69
**Fasting glucose (mg/dl)**	118.79±7.49	127.00±15.28	115.84±9.87	117.11±6.41	119.46±8.23	129.44±16.30	97.13±6.45	131.25±18.17	130.44±12.25	121.50±13.34
**HbA1c (%)**	6.35±0.19	6.82±0.69	6.47±0.19	6.42±0.13	6.58±0.25	6.30±0.29	5.85±0.169	6.17±0.18	6.23±0.16	6.08±0.13
**ALT (U/L)**	24.42±7.56	22.33±3.00	32.16±5.11	28.82±4.35	28.97±3.62	23.88±5.33	23.25±4.34	21.15±2.28	22.36±2.65	21.75±2.01
**AST (U/L)**	22.21±4.67	20.08±1.92	27.96±3.51	25.48 ±2.83	25.41±2.50	23.69±5.04	22.63±1.94	19.75±1.20	21.50 ±2.32	20.57±1.03
**BUN (mg/dl)**	15.84±0.89	15.00±1.10	16.20 ±0.80	16.05 ±0.59	15.81±0.64	18.50±2.36	17.38±1.72	15.25±0.94	16.69 ±1.18	15.86±0.83
**Creatine (mg/dl)**	0.81±0.03	0.99±0.09	0.97±0.05	0.90 ±0.03	0.97±0.04	0.94±0.06	0.84±0.11	0.81 ±0.05	0.87 ±0.04	0.82±0.04
**WBC (10**^**3**^**μl)**	8.18 ±0.45	8.00±0.72	8.86±0.62	8.57 ±0.40	8.58 ±0.48	7.59 ±0.61	7.54±0.98	8.37 ±0.54	8.02 ±0.41	8.13 ±0.47
**Platelet (10**^**3**^**μl)**	249.16±16.25	212.17 ±22.71	244.76±12.12	246.66±9.72	234.19 ±11.12	245.44 ±17.70	257.50 ±26.22	250.55 ±15.69	248.27 ±11.58	252.54±13.23

Values are given as mean ± SD. Statistical analyses for the intergroup significance between alleles and other parameters were performed by nonparametric Mann–Whitney *U* test. ANOVA test was used for intergroup significance between genotypes and other parameters. Statistical significance is shown in bold. *p*>0.05 for all

*CAE* coronary artery ectasia, *TGF β*1 transforming growth factor beta 1, HgA1c hemoglobin A1c, BUN blood urea nitrogen, WBC white blood cell, G allele (GG+GA), A allele (AA+GA), n number of samples

The association of *TGF-β1* rs1800470 (A>G) genotypes with clinical and biochemical features is shown in Table 5. Serum levels of TGF-β1 were observed to be lower in CAE patients with the common AA genotype than in heterozygous AG carriers (*p*=0.030). Additionally, CAE patients with the rare G allele had higher TGF-β1 levels than those with AA genotype (*p*=0.012). We also observed that the blood platelet count was higher in CAE patients with the common A allele (AA+AG) than in patients with the GG genotype (*p*=0.018). Serum aspartate aminotransferase (AST) levels were significantly lower in A allele carriers (AA+AG) in controls than in individuals with the GG genotype (*p*=0.019).

**Table 5:** Association between *TGF β1* rs1800470 genotype/alleles and clinical/hematological parameters in study groups

Parameters	Groups									
	CAE patient group					Control group				
	rs1800470 genotype			rs1800470 alleles		rs1800470 genotype			rs1800470 alleles	
	AA (***n***=23)	GG (***n***=10)	AG (***n***=23)	A Allele (***n***=46)	G Allele (***n***=33)	AA (***n***=13)	GG (***n***=8)	AG (***n***=23)	A Allele (***n=***36)	G Allele (***n***=31)
**Age (years)**	63.52±2.10	61.60±3.25	61.22±1.89	62.37±1.41	61.33±1.61	58.38±3.36	64.25±1.83	59.35±2.79	59.00±2.13	60.61±2.14
**TGFβ1 (ng/ml)**	2691.90±330.47 ***p=*** **0.030**	3592.50±351.49	3700.00±333.25	3183.65±245.05	3669.28±255.07 ***p*=0.012**	4293.63±998.47 ***p=*** **0.030**	4796.66±589.16	5255.00±951.77	4863.33±688.84	5130.00±703.63
**Fasting glucose (mg/dl)**	117.91±7.07	123.70±18.14	118.61±10.36	118.26±6.20	120.15±8.92	130.46±19.803	101.25±7.95	129.00±15.96	129.53±12.29	121.84±12.14
**HbA1c (%)**	6.32±0.16	6.99±0.82	6.47±0.21	6.40±0.13	6.63±0.28	6.44±0.35	5.79±0.16	6.13±0.16	6.24±0.16	6.04±0.12
**ALT (U/L)**	24.52±6.26	23.20±3.45	32.17±5.57	28.35±4.18	29.45±4.05	26.31±6.53	27.88±5.53	18.52±1.23	21.33±2.50	20.94±1.79
**AST (U/L)**	22.96±3.93	18.70±1.49	28.13±3.78	25.54±2.72	25.27±2.76	23.69±5.75	23.88±1.72	19.83±1.73	21.22±2.32	20.87±1.38
**BUN (mg/dl)**	16.00±0.84	15.80±1.28	15.65±0.79	15.83±0.57	15.70±0.66	18.15±1.70	18.13±1.85	15.61±1.55	16.53±1.17	16.26±1.25
**Creatine (mg/dl)**	0.85±0.04	0.99±0.10	0.96±0.05	0.90±0.03	0.96±0.04	0.91±0.06	0.91±0.12	0.82±0.04	0.85±0.04	0.84±0.04
**WBC (10**^**3**^**μl)**	8.50±0.49	8.80±1.01	8.23±0.56	8.37±0.37	8.40±0.49	7.50±0.48	7.65±0.98	8.27±0.58	7.99±0.41	8.11±0.49
**Platelet (10**^**3**^**μl)**	244.22±15.99	197.19±20.52	252.30±11.68	248.26±9.81 ***p*= 0.018**	235.82±10.99	247.15±20.09	249.38±30.67	251.74±13.50	250.08±11.11	251.13±12.48

Values are given as mean ± SD. Statistical analyses for the intergroup significance between alleles and other parameters were performed by nonparametric Mann–Whitney *U* test. ANOVA test was used for intergroup significance between genotypes and other parameters. Statistical significance is shown in bold

*CAE* coronary artery ectasia, *TGF β1* transforming growth factor beta 1, *HgA1c* hemoglobin A1c, *BUN* blood urea nitrogen, *WBC* white blood cell, *G Allele* (GG+GA genotypes), *A Allele* (AA+GA genotypes), *n* number of samples

**Fig. 1:**
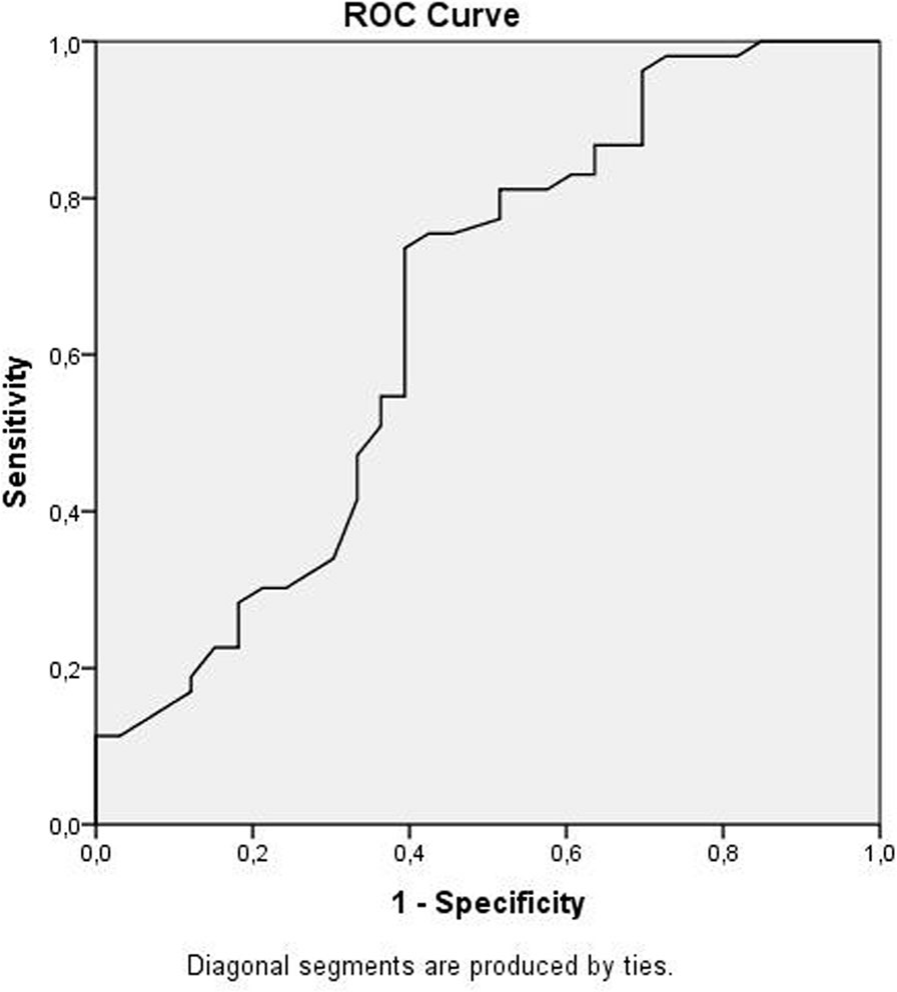
Receiver operating characteristics curve analysis of TGF-β1 levels to CAE

ROC analysis of the diagnostic accuracy of TGF-β1 in CAE patients is shown in Fig. 1. The AUC value of serum TGF-β1 levels for predicting CAE was 0.64 (95% CI 0.53–0.78 *p*=0.01). TGF-β1 blood level <3980 mcg/dL had 74% sensitivity and 61% specificity for predicting CAE.

## Discussion

The incidence of CAE was rare, at approximately 2% in our cohort, and the incidence based on previous studies ranged from 1.4 to 5.3% that were conducted in Europe and the USA. Therefore, our findings are consistent with the literature [1–4].

To the best of our knowledge, the present study is the first to show the relationship of TGF-β1 gene variations with the clinical/biochemical parameters and serum TGF-β1 levels in patients with CAE. In the present study, serum TGF-β1 levels were significantly lower in CAE patients than in controls and based on ROC analysis the discriminatory power seems to be moderate. Moreover, genotype distributions of *TGF-β1* rs1800469 and rs1800470 polymorphisms were not significantly different between the CAE and control groups. However, the common homozygous AA genotype of the *TGF-β1* rs1800470 polymorphism had lower serum TGF-β1 levels than patients with the rare G allele carriers (GG+AG genotypes) in the CAE group (*p*=0.012). In addition, we observed that the *TGF-β1* rs1800469 polymorphism was associated with serum TGF-β1 levels, which were increased in CAE patients the following order: GG < GA < AA. However, the increases were not statistically significant. Lower TGF-β1 blood levels may increase the risk of CAE development. Considering the low TGF-β1 levels in the CAE group in this study, we believe that having the *TGF-β1* rs1800470 G allele (GG+AG genotypes) and *TGF-β1* rs1800469 A allele (AA+AG genotypes) may have a protective effect against the development of CAE.

The main event in CAE pathogenesis is destruction of the ECM by arterial remodeling that is caused by serine proteinase, lysosomal proteinase, and metalloproteinases [4, 5, 12]. Because TGF-β maintains vessel wall integrity, reduces inflammation, and maintains the ECM content in atherosclerosis, when any of its effects on endothelial cells are missing, the atherosclerosis process accelerates. The role of TGF-β signaling in the pathogenesis of cardiovascular diseases has been reported previously [6, 8, 9]. In these studies, an inverse relationship between serum TGF-β1 levels and the development of atherosclerosis was demonstrated, and it was suggested that TGF-β1 concentration is severely suppressed in advanced atherosclerosis [8, 9]. In addition, Tashiro et al. demonstrated that the TGF-β plasma concentration has a prognostic significance in patients with proven CAD. In this study, the low plasma concentration TGF-β group had a poor prognosis of survival without cardiovascular events and survival without coronary interventions compared to the high plasma concentration TGF-β group [13].

These results show that TGF-β is a protective cytokine. Although there is no study examining the TGF-β blood levels in patients with CAE in the literature, the low plasma TGF-β concentrations that were found in CAE patients in our study may cause the increased risk of CAE development. In contrast to our findings, Yetkin et al. reported that plasma TGF-β1 levels were significantly increased in patients with CAE and CAD compared to age- and sex-matched patients with CAD alone [14]. In our study, the control group comprised individuals with normal coronary arteries. Therefore, the difference between the findings in the two studies could be attributed to different characteristics of the control group.

A few gene polymorphism studies on the atherosclerotic origin of CAE have been reported. These studies demonstrated the relationship between CAE and the c.894 G>T polymorphism in the endothelial nitric oxide synthase gene, HOGG1, Ser326Cys gene polymorphism, deletion in angiotensin I converting enzyme gene, and the HLA-DR B1, DR16, DQ2, and DQ5 genotypes [15–18]. Furthermore, many studies have reported the effect of *TGF-β1* gene polymorphism on atherosclerotic cardiovascular or other cardiac diseases. In a significant part of these studies, *TGF-β1* rs1800469 and rs1800470 polymorphisms were associated with restenosis after a coronary stent and coronary artery disease [19, 20]. Additionally, mutation of TGF-β signal pathway components such as TGFBR1 and TGFBR2 is directly involved in the progression of the aortic aneurysm [21]. Zuo et al. indicated the increased risk of abdominal aortic aneurysm for individuals with the *TGF-β1* rs1800469 TT(AA) genotype compared with those with the CC(GG) genotype [22]. No studies have investigated the relationship between TGF-β1 gene variations and CAE. In our study, we investigated the relationship between *TGF-β1* rs1800469 and rs1800470 polymorphisms and CAE. No significant relationship was found between *TGF-β1* polymorphisms at rs1800469 and rs1800470 and CAE. With these results, a single polymorphism alone may not be responsible for its etiology, especially considering the multidimensional presentation of the disease. Although several polymorphisms have been identified, the exact polymorphism that describes the etiology of CAE remains uncertain.

Rs1800470 is located within the *TGF-β1* gene exons, affecting the amino acid chance of developing Leu10Pro. Proline substitution at codon 10 may result from an altered intercellular signaling network or increased transcription. This may cause changes in the chemical properties and structure of the *TGF-β1* protein [11]. In CAD patients, the T(A) allele of *TGF-β1* rs1800470 (29T/C) was reportedly associated with low serum TGF-β1 levels compared to homozygous CC(GG) [23]. These findings may demonstrate the anti-inflammatory effect of *TGF-β1* on the vessel wall. In addition, Fragoso et al. demonstrated the *TGF-β1* rs1800470 (29T/C) polymorphism was related to restenosis after coronary stenting. It was also shown in the same study that individuals with the TT haplotype *TGF-β1* rs1800470 (29T/C) produced less *TGF-β1* [19]. Similar to these studies, we observed that serum TGF-*β1* levels may differ in patients with CAE according to the *TGF-β1* rs1800469 and rs1800470 polymorphisms. In the present study, homozygous AA genotype of the *TGF-β1* rs1800470 polymorphism had lower serum TGF-β1 levels than patients with the G allele carriers (GG+AG genotypes) in the CAE group. This may lead to a decrease in the anti-inflammatory effect and an acceleration in the development of CAE.

Rs1800469 is a single nucleotide polymorphism that is located within the promoter region of the *TGF-β1* gene. This mutation causes a change in the amount of *TGF-β1* that is produced without changing the protein structure. The T allele of *TGF-β1* rs1800469 was shown to be associated with a higher serum *TGF-β1* level [24]. Cao et al. found that the −509T allele (*TGF-β1* rs1800469 A allele) was associated with higher TGF-β1 expression and more severe interstitial fibrosis. Based on their findings, they suggested that increased TGF-β1 expression by the −509T allele may cause overproduction of extracellular matrix components, resulting in progressive atrial augmentation, fibrosis, and susceptibility to lone atrial fibrillation [25]. In the present study, we observed that the *TGF-β1* rs1800469 polymorphism was associated with serum TGF-β1 levels, which were increased in the following order in CAE patients: GG < GA < AA. However, the increases were not statistically significant.

The CAE group in the present study comprised individuals who had CAE with atherosclerosis. Studies have revealed that CAE is a variant of atherosclerosis and has similar pathogenesis. In this situation, it would not be a surprise to have similar risk factors. In our study, the prevalence of hypertension, hyperlipidemia, and male sex were higher in patients with CAE than in controls. Similar to our findings, Gunes et al. found in their study that the prevalence of hypertension and hyperlipidemia, which are clinical features of CAE patients, was more frequent in CAE patients than in controls who had normal coronary arteries [26]. These are also well-known risk factors for the development of CAD. Moreover, Kamal et al. also found in their study that male gender was frequent in patients with CAE and an independent risk factor for CAE development [27].

### Limitations

This was a single-center study with a relatively small number of patients. In addition, there was no long-term follow-up.

## Conclusion

Thus, TGF-β1 levels were significantly lower in CAE patients than in controls in our study. These results suggest that TGF-β1 may have a protective effect against CAE development. Furthermore, *TGF-*β1 rs1800470 G allele carriers were shown to have higher TGF-β1 levels than patients with the AA genotype in the CAE group. This suggests that having the G allele in the *TGF-β1* rs1800470 polymorphism could prevent CAE development. Considering the higher prevalence of hyperlipidemia and hypertension that is found in patients with CAE in our study, strict control of hyperlipidemia and hypertension may be a protective strategy against CAE development

## Data Availability

All data generated or analyzed during this study are included in this published article.

## Abbreviations

CAE: Coronary artery ectasia
USA: United State of America
TGF-β: Transforming growth factor beta
ECM: Extracellular matrix
SNP: Single nucleotide polymorphism
DNA: Deoxyribo nucleic acid
ROC: Receiver operating characteristic
AUC: Area under curve
HWE: Hardy–Weinberg equation
CAD: Coronary artery disease
